# Editorial: Asthma and mental health: novel insights to the experience, etiology, longitudinal course, and management of mental health in asthma and allergy

**DOI:** 10.3389/falgy.2025.1559527

**Published:** 2025-02-17

**Authors:** Chris Barton, Lorraine Smith, Jean-Marie Bruzzese

**Affiliations:** ^1^ School of Public Health and Preventive Medicine, Monash University, Melbourne, VIC, Australia; ^2^ Faculty of Medicine and Health, University of Sydney, Sydney, NSW, Australia; ^3^ Columbia University School of Nursing, New York, NY, United States

**Keywords:** asthma, mental health, asthma control, self-management, social determinant, quality of life

**Editorial on the Research Topic**
Asthma and mental health: novel insights to the experience, etiology, longitudinal course, and management of mental health in asthma and allergy

Mental health problems are common co-occurring conditions with asthma and allergies that impact self-management and well-being across the life course. Understanding of the etiology, epidemiology and impact of these on people living with asthma and allergy has grown markedly over the past 25 years. The bi-directional relationship between asthma, anxiety and depressive disorders is well established (1–4), with the likelihood of mental health problems increasing as a function of disease severity (2, 5). Perceived stress and emotional difficulties may exacerbate the physical symptoms and severity of existing allergic conditions and contribute to an increased need for medical care.

This Research Topic presents four papers that offer a new and deeper understanding of the relationship between asthma and mental health. They include studies of caregivers of preschool children with asthma and youth, adolescents and adults living with asthma. They encompass articles reporting novel insights and links between asthma, allergy and mental health, and how mental health and emotions can be managed to enhance self-management and support the well-being and quality of life of individuals living with asthma.

The works submitted go beyond the individual to consider the social determinants of good asthma control and self-management. Hiles et al. considered the extent to which social determinants relate to mental health in people with asthma, highlighting the importance of health inequities to inform population-level preventive strategies for asthma and mental health. They showed that age, sex and employment/student status are associated with components of mental health in adults with asthma and contribute to a growing body of work calling for a holistic approach to health and health equity in people with asthma, particularly those who experience co-occurring mental health problems.

Asthma self-management is essential for asthma control and can be negatively impacted by depression. Patel et al. used baseline data from a randomized trial of an asthma intervention for adolescents in rural areas of South Carolina, finding that more than half of the participants (61.4%) were at risk for depression. Also, depressive symptoms were significantly related to asthma control, such that as depressive symptoms increased, asthma control declined. In contrast, depressive symptoms were not associated with asthma self-management in this study, suggesting that self-management, at least those aspects assessed by the authors, is not an avenue by which depression impacts asthma control in adolescents.

Parents are also important in the management of asthma, and caring for a child with asthma can pose risks to the mental health of caregivers. Because children rely on caregivers to manage medication administration, identifying families who are at risk for poor medication adherence is important to improve asthma outcomes. Lu et al. investigated the association between asthma routines, family asthma management knowledge and skills, and caregiver depressive symptoms with daily controller medication adherence among Head Start preschool children in Baltimore City. The authors found that nearly a quarter of caregivers (24%) had clinically significant depressive symptoms and demonstrated that better medication routines and family asthma management were associated with higher medication adherence to daily asthma controller medications in a low-income, urban, preschool group.

Recent experiences of people living with asthma have included the need for special care with the emergence of COVID-19. Respiratory viruses are a common asthma trigger. Regulatory guidelines indicated that individuals with asthma were at higher risk for complications related to COVID-19 and as such should take additional precautions to prevent transmission of COVID-19. Paradoxically, people with chronic respiratory disease tended to have better control of their condition during the pandemic. Although there were positive asthma-related morbidity and healthcare utilization outcomes in the context of the pandemic, less was known about the relationship between pandemic onset and youth and caregiver psychosocial functioning. This was addressed in the paper by Sinisterra et al., who aimed to characterize youth asthma exacerbations, control, and quality of life across three distinct phases of the COVID-19 pandemic, and to describe caregiver asthma-related quality of life in this context. The researchers confirmed previous reports of better overall asthma functioning during the pandemic. Despite this, no differences in asthma-related quality of life were found between the distinct phases of the pandemic for youth living with asthma and ratings of youth asthma-related quality of life remained steadily high, on average, regardless of the time of enrollment.

The four studies published in this research topic highlight the interplay between family dynamics, routines, and mental health in managing asthma. They emphasize their interconnectedness and offer valuable insights for healthcare providers and families. Collectively, they advance evidence for the need to shift toward a more holistic approach to asthma management. They suggest that interventions should focus on strengthening family support systems, educating families about asthma management, and addressing mental health concerns in both patients and their caregivers. Additionally, recognizing and mitigating the impact of social determinants of health is crucial to improving asthma outcomes, especially for disadvantaged communities.

As our understanding of the interplay between mental health conditions and asthma grows, future research should focus on developing and evaluating multifaceted interventions that address the interconnectedness of family dynamics, routines, mental health, and social determinants of health in asthma management. These could include tailored education, skills training, social support, and collaborative models of care. By taking a more holistic and integrated approach to asthma research and interventions, we can further improve the lives of people living with asthma and their families, across the life course.

## References

[1] Jang S. Temporal and bidirectional association of depression and physical illnesses: analyzing the pooled data from independently conducted cross-sectional national surveys at three distinct time points. J Psychosom Res. (2024) 179:111614. 10.1016/j.jpsychores.2024.111614 38422716

[2] Dave ND Xiang L Rehm K Marshall G Jr. Stress and allergic diseases. Immunol Allergy Clin North Am. (2011) 31(1):55–68. 10.1016/j.iac.2010.09.009 21094923 PMC3264048

[3] Chida Y Hamer M Steptoe A. A bidirectional relationship between psychosocial factors and atopic disorders: a systematic review and meta-analysis. Psychosom Med. (2008) 70:102–16. 10.1097/PSY.0b013e31815c1b71 18158379

[4] Slattery M. Psychiatric comorbidity associated with atopic disorders in children and adolescents. Immunol Allergy Clin North Am. (2005) 25:407–20. 10.1016/j.iac.2005.02.007 15878463

[5] Goodwin R Robinson M Sly P McKeague I Susser E Zubrick S Severity and persistence of asthma and mental health: a birth cohort study. Psychol Med. (2013) 43(6):1313–22. 10.1017/S0033291712001754 23171853 PMC3857579

